# Transcriptome sequencing reveals distinct atypical parathyroid tumor subtypes

**DOI:** 10.1530/ERC-25-0057

**Published:** 2025-06-14

**Authors:** Hye-Sun Park, Milim Kim, Se-Young Jo, Gi Jeong Kim, Jong Ju Jeong, Namki Hong, Sangwoo Kim, Yumie Rhee

**Affiliations:** ^1^ Department of Internal Medicine, Gangnam Severance Hospital, Yonsei University College of Medicine, Seoul, Korea; ^2^ Department of Pathology, Yonsei University College of Medicine, Seoul, Korea; ^3^ Department of Biomedical Systems Informatics, Yonsei University College of Medicine, Seoul, Korea; ^4^ Department of Genetics and Genomics, Boston Children's Hospital, Boston, Massachusetts, USA; ^5^ Department of Pediatric, Harvard Medical School, Boston, Massachusetts, USA; ^6^ Department of Pathology, Hallym Hospital, Incheon, Korea; ^7^ Department of Surgery, Thyroid Cancer Clinic, Yonsei University College of Medicine, Seoul, Korea; ^8^ Department of Internal Medicine, Severance Hospital, Endocrine Research Institute, Yonsei University College of Medicine, Seoul, Korea

**Keywords:** atypical parathyroid tumor, parathyroid cancer, parathyroid adenoma, malignancy risk, transcriptome analysis

## Abstract

Atypical parathyroid tumors (APTs) are a rare subtype of parathyroid neoplasms characterized by diagnostic challenges and an uncertain prognosis. This study aimed to validate the subtypes of APTs using transcriptome sequencing. We applied a clustering model developed for our previous study in which we had successfully distinguished parathyroid cancer from adenomas using gene expression patterns. Sixteen patients with APT who had undergone parathyroidectomy were enrolled, and we analyzed their baseline data, pathologic reports and follow-up records and performed transcriptome sequencing of their APT samples. We then used our clustering model to classify tumors as either cancer- or adenoma-type APTs and compared these results with clinical findings. The median age of patients was 48.9 years, with median calcium and parathyroid hormone (PTH) levels of 11.4 mg/dL and 420.0 pg/mL, respectively. Pathologic and immunohistochemical results did not reveal any remarkable differences between adenoma-type and cancer-type APTs. However, clustering analysis classified four of the 16 APTs as being cancer-type and 12 as being adenoma-type tumors. Cancer-type patients had a median age of 30.0 years, with median calcium and PTH levels of 12.6 mg/dL and 800.8 pg/mL, respectively, clinically resembling parathyroid cancer. One patient exhibited a somatic *CDC73* two-hit mutation and positive WT1 staining, suggesting a high malignant potential. Clustering analysis through transcriptome sequencing shows promise for risk stratification of patients with APTs. For those classified as having cancer-type tumors, close monitoring and long-term follow-up may be warranted.

## Introduction

Atypical parathyroid tumors (APTs) are a rare form of parathyroid tumor (Galani *et al.* 2021). While most cases of primary hyperparathyroidism (PHPT) arise from adenomas (80–85%) or hyperplasia (10–15%), APTs account for approximately 1%, similar to the incidence of parathyroid cancer (1%) (DeLellis 2011, Galani *et al.* 2021). Despite their rarity, APTs hold clinical significance as an intermediate entity between parathyroid adenomas and parathyroid cancers, leading to an uncertain malignant potential. Although the molecular pathogenesis of APTs remains unclear, germline CDC73 mutations have been identified in a subset of cases (Saponaro *et al.* 2021). In addition, recent molecular studies have shown that APTs share genomic alterations with both benign and malignant parathyroid tumors, supporting their classification as an intermediate entity along the adenoma-carcinoma spectrum (Pardi *et al.* 2024).

These molecular findings are consistent with the histological and biochemical characteristics that place APTs between parathyroid adenomas and carcinomas. APTs share atypical cytological and architectural features that are seen in parathyroid cancer, such as band-like fibrosis, trabecular growth patterns, atypical mitotic figures and nuclear atypia, but do not exhibit unequivocal invasion such as lymphovascular or perineural invasion (Cetani *et al.* 2019, Erickson *et al.* 2022). Moreover, laboratory findings of APTs, including calcium and parathyroid hormone (PTH) levels, fall between those of parathyroid cancers and parathyroid adenomas (Christakis *et al.* 2016, Galani *et al.* 2021). A single-center study reported that most APT patients had favorable outcomes despite presenting with more severe biochemical profiles than those with adenomas (Saponaro *et al.* 2021). However, some cases of recurrences or metastases similar to parathyroid cancer have been reported (Galani *et al.* 2021, Barale *et al.* 2023). Given the poor prognosis of parathyroid cancer, it is crucial to identify cases that mimic its clinical course (Betea *et al.* 2015).

Several immunohistochemical (IHC) staining markers have been developed to predict the malignant potential of parathyroid tumors. The most well-known marker is parafibromin, with others including protein gene product 9.5 (PGP9.5), galectin-3, p53 and Ki-67 (Sharretts *et al.* 2010, Kruijff *et al.* 2014, Truran *et al.* 2014). However, the low sensitivities and specificities of these markers limit their clinical use. In our previous study, we performed transcriptome sequencing to successfully distinguish parathyroid cancers from parathyroid adenomas based on a differentially expressed gene (DEG) analysis (Jo *et al.* 2023). This clustering effectively distinguished parathyroid carcinomas from adenomas and aligned well with the clinical findings, demonstrating the potential utility of this approach for identifying the malignant potential of APTs. Therefore, in this study, we applied this previous clustering model to evaluate the malignant potential of APTs and to assess their alignment with clinical findings, thereby potentially validating the clinical utility of this classification model.

## Methods

### Study participants and sample acquisition

Sixteen patients with APTs who had undergone parathyroidectomy at Severance Hospital between 2012 and 2022 were enrolled. Written consent was obtained from all participants before surgery, agreeing to the preservation and secondary use of human-derived materials. Tumor samples from these patients were collected during surgery for PHPT. The tumor tissues were thoroughly reviewed, and the diagnosis of APT was confirmed by two experienced pathologists (M K and G J K) according to the 5th edition of WHO classification of tumors of endocrine organs (Erickson *et al.* 2022, WHO Classification of Tumours Editorial Board 2022). Multiple sections were examined, and a thorough evaluation was performed to ensure that these cases do not show unequivocal evidence of capsular, vascular or perineural invasion or invasion into adjacent structures, in order to rule out the possibility of parathyroid carcinoma. The detailed diagnostic criteria are summarized in Supplementary Table 1 (see section on Supplementary materials given at the end of the article).

A fraction of the samples was preserved in RNA preservative (RNAlater, Invitrogen, USA) immediately after tumor excision in the operating room. These preserved samples were then stored at −80°C in a parathyroid tissue bank until thawing for RNA extraction. The isolation of total RNA was carried out using a commercial kit (RNeasy Mini Kit, Qiagen, South Korea) with the RNA-Bee reagent (AMSbio, USA), following the manufacturer’s instructions. The concentration of RNA was measured using a Qubit Fluorometer (Thermo Fisher, USA), and the integrity of RNA was evaluated using a 4200 TapeStation (Agilent, USA). An RNA concentration of ≥3 ng/μL was considered sufficient for gene expression, and its quality was considered to be acceptable with a DV200 value of ≥70%.

To compare the baseline characteristics of patients, we established a reference group consisting of individuals with benign parathyroid adenomas who had visited Severance Hospital between 2020 and 2023 and met the following criteria: i) aged over 19 years, ii) underwent parathyroidectomy for PHPT and iii) were confirmed to have benign parathyroid adenomas based on pathological reports. This group, comprising 273 patients, was designated as the reference group. Before surgery, written consent was obtained from all patients for the secondary use of their surgical specimens for research purposes, ensuring proper deidentification according to institutional protocols. This study was approved by the Institutional Review Board of Severance Hospital (No. 4-2019-1067; Seoul, Republic of Korea).

### Clinical data and genome sequencing

Demographic and clinical data were collected from patients with APT and the reference group. The baseline measurements included calcium, phosphorus, albumin, alkaline phosphatase, intact PTH, blood urea nitrogen, creatinine, 25-hydroxy vitamin D, ionized calcium and 24 h urinary calcium levels. Albumin-corrected calcium levels were determined using the following formula: serum calcium (mg/dL) + 0.8 × (4.0 – albumin (g/dL)) (Bilezikian *et al.* 2014). The intact PTH concentration in serum was measured through a second-generation PTH assay (Elecsys PTH, Roche Diagnostics, Germany) on a Cobas e801 immunoassay analyzer (Roche Diagnostics). Bone mineral densities (BMDs) of the lumbar spine, femoral neck, total hip and one-third radius were measured using dual energy X-ray absorptiometry (Discovery W, Hologic, Inc., USA). Abdominal sonography and computed tomography (CT) images were reviewed for detection of urinary tract stones. Follow-up biochemical data were collected after surgery, and recurrence was defined as elevated serum calcium and/or PTH levels occurring at least 6 months after initial postoperative normocalcemia (Guerin *et al.* 2017).

Genomic analysis data, collected retrospectively from prior medical records, were available for 12 of the 16 enrolled patients. Genomic sequencing was performed during clinical evaluation in patients who presented with atypical features such as young age, male sex or relatively large tumor size (Lassen *et al.* 2014, Park *et al.* 2022). These analyses were conducted using either targeted gene sequencing, which included 400 genes associated with endocrine disorders, or clinical exome sequencing, which included 4,503 genes on the xGen Inherited Diseases Panel (Integrated DNA Technologies, USA). Genomic DNA was extracted from leukocytes in peripheral blood samples utilizing the QIAamp Blood DNA Mini Kit (Qiagen, Germany), as per the manufacturer’s instructions. Subsequent sequencing and data analysis were performed as previously described (Rim *et al.* 2018, Kim *et al.* 2019). Variant interpretation followed the 5-tier classification system recommended by the American College of Medical Genetics and Genomics and the Association for Molecular Pathology guidelines (Richards *et al.* 2015).

### Reference molecular classification model

In our previous study, we used 49 parathyroid tissues (11 carcinomas, 28 adenomas and 10 normal tissues) to identify cancer-specific genes that differentiate parathyroid cancer from adenomas and normal parathyroid tissue (Jo *et al.* 2023). Based on the expression levels of these genes, we conducted an unsupervised hierarchical clustering, which was able to identify cancer samples with high accuracy. In the current study, transcriptome sequencing was performed for the 16 APT samples, and their expression data were analyzed using the 597 carcinoma-specific DEGs identified in our previous study (Jo *et al.* 2023). Gene expression profiles of parathyroid carcinoma, adenoma and normal tissues were obtained from the prior dataset and used for comparison and clustering. The model was constructed through the following process: first, RNA-seq data aligned to the GRCh38 human genome reference with the STAR2 (v2.7.8a) aligner (Dobin *et al.* 2013) were processed with featureCounts to count reads mapped to 19,504 known gene regions. These raw read counts were then processed using the DESeq package in R and transformed using variance stabilizing transformation (VST). Based on the VST values, the ViDGER (v.1.10.0) package’s vsFourWay() function was used to identify 597 carcinoma-specific DEGs (McDermaid *et al.* 2019). Unsupervised hierarchical clustering using the VST values of these 597 genes confirmed the model’s capacity to classify carcinoma samples exclusively. Further details on this process are provided in our previous study (Jo *et al.* 2023).

### Generation and preprocessing of sequencing data

Transcriptome sequencing was performed on the 16 APT tissues from 16 individuals. These samples passed the quality check and were sequenced using the Illumina Total RNA Sequencing (Illumina, USA) library. All RNA sequencing data were aligned to the genome index generated from the GRCh38 reference genome and GENCODE (v33) annotation using the STAR2 (v2.7.8a) aligner (Dobin *et al.* 2013). Sequencing duplicates were excluded using MarkDuplicates of the GATK (v4.0.1.1) toolkit (van der Auwera & O'Connor 2020). In addition, whole-exome sequencing (WES) with a target depth of 200X was performed on a sample suspected to have a double somatic mutation in *CDC73* (patient 9). Raw reads from the WES were aligned to the GRCh38 reference genome using the BWA-MEM aligner (v0.7.17-r1188) (Li 2013). Preprocessing steps, including MarkDuplicates and FixMateInformation, were carried out using GATK (v4.0.1.1). The data generated in this study are publicly available in NCBI SRA at PRJNA1184316.

### Confirming variants on CDC73

As only RNA-seq data were available in this study, we identified and verified small variants from these data using the ‘RNA mode’ of Strelka2 (v2.9.10) (Kim *et al.* 2018). We applied SplitNCigarReads of GATK (v4.0.1.1) to the BAM files to split all intron-skipping aligned reads. Then the RNA mode of Strelka2 (--rna argument) was applied to the BAM file to call SNVs and INDELs. The variants called within the region of the cell division cycle (*CDC73*) gene were further manually inspected using the Integrative Genomes Viewer (IGV, v11.0.7). For patient 9, who had two somatic mutations called, we confirmed this rare case by also verifying the WES data from the same tissue in parallel with IGV and confirmed the presence of both somatic mutations.

### Immunohistochemical staining

We performed IHC staining using previously studied markers for parathyroid cancer, including parafibromin, galectin-3, Ki-67, PGP9.5 and p53 (Truran *et al.* 2014, Erickson & Mete 2018). In addition, Wilms tumor 1 (WT1) staining was included, as our previous study using whole transcriptome sequencing data suggested it could be a marker for *CDC73*-mutant parathyroid cancer (Jo *et al.* 2023). The formalin-fixed, paraffin-embedded tissue blocks were cut into 4 μm sections. IHC staining was performed using a Ventana XT automated stainer (Ventana Medical System, USA) for parafibromin, galectin-3, PGP9.5, p53, Ki-67 and WT1. The following antibodies were used: parafibromin (clone 2H1; 1:50, Santa Cruz Biotechnology, USA), galectin-3 (clone 9C4; ready to use; Leica Biosystems, Germany), PGP9.5 (polyclonal; 1:100, CellMarque, USA), p53 (clone DO7; 1:300, Novocastra, UK) and WT1 (clone 6F-H2, 1:100; CellMarque) according to the manufacturer’s instructions. IHC staining was evaluated using light microscopy. For evaluation of parafibromin, a complete loss of nuclear expression in all tumor cells was considered to be ‘parafibromin-deficient’ (Erickson *et al.* 2022). Galectin-3 and PGP9.5 were considered positive when the cytoplasm was globally or focally stained. Diffuse nuclear staining or a complete loss of p53 was considered to be aberrant expression patterns. WT1 expression was graded as either weak, moderate or strong based on the intensity of nuclear expression. Tissues exhibiting WT1 expression in <5% of tumor cells of any intensity grade or those with a weak intensity were regarded to be negative, whereas tissues showing moderate to strong intensities in ≥5% of tumor cells were regarded to be positive for WT1 expression, as described in our previous study (Jo *et al.* 2023) and these results were confirmed by two endocrine pathologists (M K and G J K). For the evaluation of Ki-67 L.I, the whole-section immunostained slides were scanned, and hotspots were manually selected by pathologists for digital quantification (PACS, Sectra AB, Sweden).

### Statistical analysis

Continuous variables are presented as medians with interquartile ranges based on normality tests, including both Q–Q plots and the Shapiro–Wilk test. Categorical variables are presented as numbers with percentages (%). Intergroup comparisons were performed using the Kruskal–Wallis test with Dunn’s test for non-normally distributed continuous variables, while categorical variables were analyzed using the Fisher’s exact test. A linear regression analysis was performed to examine the relationship between serum PTH levels and the longest diameter of the tumor, stratified by pathology group (cancer-type APTs, adenoma-type APTs and reference group), and a corresponding scatter plot was generated. To assess the association between serum PTH levels and BMD, both Pearson correlation and linear regression analyses were conducted. *P*-values <0.05 were considered statistically significant. All statistical analyses were performed using R software 4.2.2 (https://www.R-project.org).

## Results

**Figure 1:**
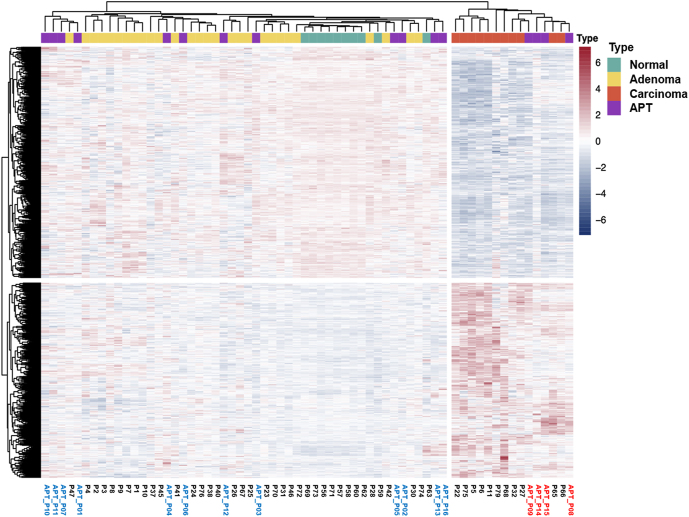
Classification of APTs based on hierarchical clustering. Hierarchical clustering result of 65 parathyroid tumor patients based on 597 carcinoma-specific DEGs. Sixteen APT patients were classified into two subtypes: cancer-type (blue) and adenoma-type (red).

Baseline characteristics of the 16 patients are presented in Table 1. The median age of patients was 49.5 (36.0–62.5) years, and 62.5% were women. The median levels of serum corrected calcium and PTH were 11.4 (10.3–13.1) mg/dL and 420.0 (95.5–777.8) pg/mL, respectively. Genomic sequencing was performed on peripheral blood samples from 12 participants, with all results classified as variants of uncertain significance (VUS). All patients underwent parathyroidectomy for PHPT and were confirmed to have no family history of parathyroid or other endocrinologic tumors based on clinical interviews, indicating that all cases were sporadic. Focused parathyroidectomy was performed based on preoperative imaging findings. Intraoperative PTH monitoring was used to assess the adequacy of resection, with surgical success defined as a >50% decrease in PTH from the highest pre-incision or pre-excision level measured 10 min after lesion removal (Carneiro *et al.* 2003). Postoperative success was further evaluated by normalization of calcium and PTH levels in accordance with previous studies (Wilhelm *et al.* 2016, Bilezikian *et al.* 2022). The pathologic review confirmed that all parathyroid tumor samples from the 16 patients were APTs (Supplementary Table 1 and Supplementary Fig. 1). After the clustering analysis using transcriptome sequencing, 12 samples were classified as adenoma-type APTs whereas four samples (patients 8, 9, 14 and 15) were classified as cancer-type APTs (Fig. 1).

**Table 1:** Baseline characteristics of study participants.

		Patients with APTs			Reference group (adenoma) (*n* = 273)	*P*-value
		Total (*n* = 16)	Cancer-type (*n* = 4)	Adenoma-type (*n* = 12)		
Age		49.5 (36.0–62.5)	30.0 (25.5–33.0)*^,^^†^	56.5 (47.0–64.5)	59.6 (48.9–66.2)	0.004
Women, *n* (%)		10 (62.5%)	2 (50.0%)	8 (66.7%)	211 (77.3%)	0.317
Corrected calcium, mg/dL		11.4 (10.3–13.1)	12.6 (11.3–13.9)^†^	10.9 (10.2–12.3)	10.6 (10.1–11.1)	0.012
Phosphate, mg/dL		2.3 (2.2–2.8)	2.2 (1.9–2.5)	2.5 (2.3–2.8)	2.8 (2.4–3.1)	0.014
BUN, mg/dL		14.0 (11.3–18.2)	14.0 (9.6–16.3)	14.0 (11.3–19.4)	14.5 (11.9–17.9)	0.817
Creatinine, mg/dL		0.8 ± 0.3	0.7 (0.6–0.9)	0.8 (0.6–1.0)	0.7 (0.6–0.9)	0.971
PTH, pg/mL		420.0 (95.5–777.8)	800.8 (640.8–865.5)*^,^^†^	134.8 (93.5–550.0)	123.0 (93.2–176.5)	0.003
Ionized calcium, mg/dL		6.0 (5.6–6.6)	6.5 (6.0–6.7)^†^	5.7 (5.4–6.5)	5.5 (5.3–5.8)	0.008
25OHD, mg/dL		13.3 (9.3–18.4)	9.3 (8.4–12.7)^†^	15.1 (10.8–24.2)	19.4 (12.8–27.4)	0.021
24U calcium		315.1 (200.6–348.6)	341.6 (280.3–451.0)	213.9 (177.5–336.2)	289.6 (211.2–388.2)	0.453
BMD T- score	LS	−1.9 (−2.6–−0.1)	−1.6 (−3.1–−0.1)	−1.9 (−2.6–−0.3)	−1.9 (−2.8–−0.9)	0.695
	FN	−1.8 (−2.6–−0.4)	−1.6 (−3.0–−0.1)	−1.8 (−2.4–−0.8)	−1.9 (−2.5–−1.2)	0.873
	TH	−1.1 (−1.8–−0.6)	−1.1 (−2.2–−0.6)	−1.1 (−1.6–−0.3)	−1.2 (−1.8–−0.4)	0.867
	1/3 radius	−4.2 (−4.5–−2.3)	−4.2 (−5.2–−4.0)	−2.8 (−4.5–−0.9)	−2.6 (−4.1–−1.3)	0.090
Tumor size	Longest diameter (cm)	1.9 (1.5–3.0)	2.4 (1.8–3.6)	1.9 (1.2–3.0)	1.5 (1.0–1.8)	0.009
	Shortest diameter (cm)	1.6 (1.1–1.8)	1.8 (1.4–2.0)^†^	1.5 (1.0–1.8)	0.5 (0.4–0.6)	0.001

* Vs adenoma-type.

^†^ Vs reference group.

*Abbreviations*: APT, atypical parathyroid tumor; BUN, blood urea nitrogen; PTH, parathyroid hormone; 25OHD, 25-hydroxy-vitamin D; 24U, 24 h urine; BMD, bone mineral density; LS, lumbar spine; FN, femur neck; TH, total hip.

To further characterize the molecular similarity of APT subtypes to classical parathyroid tumors, we compared the expression profiles of DEGs between adenoma-type APTs and classical adenomas and between carcinoma-type APTs and classical carcinomas. As shown in Supplementary Fig. 2, gene expression patterns of APT subtypes showed high concordance with their respective classical tumor types (*R* = 0.87 and 0.84, respectively). In addition, gene ontology enrichment analysis (Supplementary Fig. 3) revealed that downregulated genes in adenoma-type APTs were enriched for immune-related pathways similar to classical adenomas while carcinoma-type APTs and classical carcinomas both showed enrichment in pathways related to extracellular matrix remodeling and steroid metabolism. These findings further support the transcriptional and biological similarity of APT subtypes to classical adenoma and carcinoma, respectively.

We investigated whether the four patients with cancer-type APTs had clinical manifestations that were distinct from those who were classified as having adenoma-type APTs (Table 1). For the cancer-type patients, the median age was 30.0 (25.5–33.0) years, and two (50%) were women. The median levels of corrected calcium and PTH were 12.6 (11.3–13.9) mg/dL and 800.8 (640.8–865.5) pg/mL, respectively, both higher than in those with adenoma-type APTs (10.9 mg/dL and 134.8 pg/mL, respectively). Furthermore, compared with the reference group, patients with cancer-type APTs were significantly younger and had significantly higher levels of corrected calcium and PTH. Tumor size varied significantly across the groups, with patients in the cancer-type APT group exhibiting notably larger tumors. Although not statistically significant, the BMD T-score at the 1/3 radius was lower for patients with cancer-type APT than for those with adenoma-type APTs or the reference group. Given that cancer-type APTs were associated with larger tumor size and lower BMD, we examined whether tumor size and BMD were correlated with serum PTH levels (Supplementary Fig. 4 and Supplementary Table 2). A positive correlation between tumor size and PTH was observed in the reference group (*P* = 0.0006) but not in the APT groups, likely due to small sample sizes. In addition, higher PTH levels were significantly associated with lower BMD at multiple skeletal sites.

The age at diagnosis for the four patients with cancer-type APTs was 22, 29, 35 and 31 years, which were the youngest ages of all 16 patients (Table 2). These patients had been diagnosed with PHPT with high calcium and PTH levels, and all had renal stones confirmed either via abdominal sonography or abdominal-pelvic CT imaging. In addition, patient 8 had a history of a forearm fracture. Clinical exome sequencing of blood DNA revealed VUS with no pathogenic or likely pathogenic variants identified. Although classified as VUS, several candidate genes previously reported to be associated with parathyroid disease, including *ITPR2*, *IL21R*, *MMP14*, *TWIST1* and *ESR2* (Cetani *et al.* 2020) were identified in three patients with cancer-type tumors. Similarly, germline sequencing of blood DNA in patients classified as having the adenoma-type APTs also identified VUS. Among these, several genes such as *APC, CEP152, CREBBP, FAM111A, FGFR1, KL* and *MMP14* have been previously suggested to be associated with parathyroid disease (Cetani *et al.* 2020).

**Table 2:** Clinical findings of the four patients with cancer-type APTs.

Patient No	Age at diagnosis (years)	Sex	Corrected calcium (mg/dL)	PTH (pg/mL)	BMD Z-score at 1/3 radius	Other clinical manifestations	IHC staining		*CDC73* variant detected by transcriptome sequencing	Clinical exome sequencing of blood DNA	Clinical course after surgery
							WT1	parafibromin			
8	22	M	11.4	507.0	−3.9	Renal stone, forearm fracture	(−)	No loss	(−)	All VUS *FBN1*, ITPR2*, *ACAN*, *IFT140*, IL21R*, *CDK5RAP2*, *OBSL1*, MMP14*	No recurrence
9	29	M	14.1	774.6	−6.1	Renal stone	(+)	Loss	c.15delT (frameshift), c.376C>T (stop gained)	All VUS *TRPS1*, TWIST1*, *TGDS*, *COL27A1*, *HGSNAT*, *LFNG*, *SH3PXD2B*, *DYSF*	No recurrence
14	35	F	13.7	904.0	−4.0	Renal stone	(−)	No loss	c.34A>G (missense)	All VUS *CYP24A1*, *DCC*, *FLNB*, *COL1A2*, *GALT*, *PANK2*, *PANK2*, *APOE*	No recurrence
15	31	F	11.1	827.0	−4.1	Renal stone	(−)	No loss	(−)	All VUS ESR2*, *APOB*, *DUOX2*, *DUOX2*, *DYM*, *CEP290*	Persistent PTH elevation

* Genes that have previously been reported to be associated with parathyroid disease.

*Abbreviations*: M, Male; F, female; PTH, parathyroid hormone; BMD, bone mineral density; IHC, immunohistochemical; VUS, variants of uncertain significance; APT, atypical parathyroid tumor.

**Figure 2:**
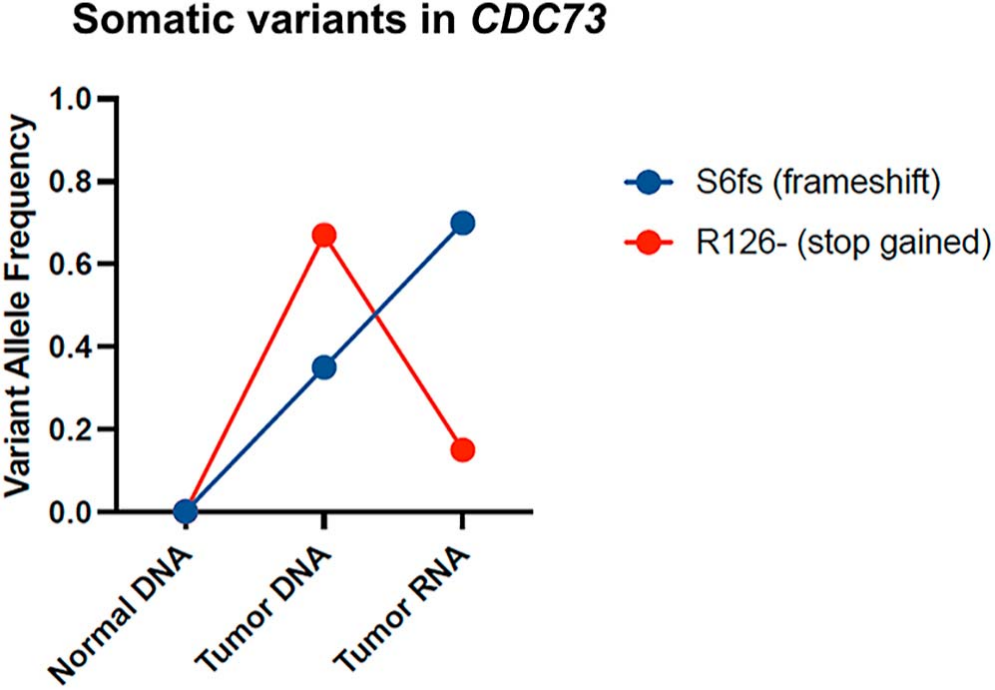
Bi-allelic somatic mutations in *CDC73* of patient 9. Variant allele frequencies (VAFs) of two CDC73 mutations, S6fs (frameshift, blue) and R126* (stop gained, red), are shown in normal DNA, tumor DNA and tumor RNA. The opposite VAF patterns in tumor RNA suggest that these mutations are on separate alleles, indicating a double somatic mutation resulting in bi-allelic loss of CDC73 in this patient’s tumor.

The *CDC73* mutation status was investigated through variant calling on transcriptome sequencing data. Patient 9, classified as having a cancer-type APT, exhibited two distinct mutations within the genomic region of *CDC73*: a frameshift mutation (c.15delT) and a stop-gained mutation (c.376C>T). Given the rarity of double somatic mutations in the *CDC73* gene, we performed WES on his tumor tissue for confirmation. Our analysis confirmed that both mutations were somatic. Notably, the opposite pattern of variant allele frequencies for these two somatic mutations in DNA and RNA suggested that they were located on opposite alleles, indicating a biallelic loss of CDC73 in this patient (Fig. 2). Patient 14, also with a cancer-type APT, had a *CDC73* missense variant (c.34A>G). Patient 3 and patient 4, whose tumors were classified as adenoma-type APTs, also had a *CDC73* missense variant (c.1582C>T and c.742A>G, respectively). However, sequencing errors cannot be ruled out in these patients because the allelic counts were extremely low.

IHC staining for parafibromin, PGP 9.5, galectin3, p53, Ki67 and WT1 was also performed (Table 3). All cancer-type APTs had positive staining for PGP 9.5 and galectin-3; however, these markers were not specific to cancer-type tumors. Ki-67 expression also varied across the samples. Loss of parafibromin staining, the most recognized IHC marker for parathyroid cancer, was observed only in patient 9, who had a cancer-type APT. In addition, the tumor of patient 9 stained positive for WT1, a novel IHC marker for *CDC73*-mutated parathyroid cancer as suggested in our previous study (Jo *et al.* 2023) (Supplementary Fig. 5).

**Table 3:** Immunohistochemical staining results of study participants.

Patient No.	Clustering	Parafibromin	WT1	PGP9.5	galectin3	p53	Ki-67
1	Adenoma	No loss	(−)	Focal positive	(−)	Nonspecific expression	1.5%
2	Adenoma	No loss	(−)	Focal positive	Focal positive	Nonspecific expression	2.5%
3	Adenoma	No loss	(−)	(−)	(−)	Nonspecific expression	0.7%
4	Adenoma	No loss	(−)	Focal positive	(−)	Nonspecific expression	5.6%
5	Adenoma	No loss	(−)	(−)	(−)	Nonspecific expression	0.5%
6	Adenoma	No loss	(−)	Focal positive	Focal positive	Nonspecific expression	2.1%
7	Adenoma	No loss	(−)	Focal positive	Focal positive	Nonspecific expression	11.2%
**8**	**Cancer**	**No loss**	**(−)**	**Focal positive**	**Positive**	**Nonspecific expression**	**2.5%**
**9**	**Cancer**	**Loss**	**Positive**	**Positive**	**Positive**	**Nonspecific expression**	**0.6%**
10	Adenoma	No loss	(−)	Focal positive	Focal positive	Nonspecific expression	2.0%
11	Adenoma	No loss	(−)	Focal positive	Focal positive	Nonspecific expression	0.7%
12	Adenoma	No loss	(−)	Positive	(−)	Nonspecific expression	0.6%
13	Adenoma	No loss	(−)	Focal positive	(−)	Nonspecific expression	7.5%
**14**	**Cancer**	**No loss**	**(−)**	**Positive**	**Focal positive**	**Nonspecific expression**	**2.2%**
**15**	**Cancer**	**No loss**	**(−)**	**Positive**	**Focal positive**	**Nonspecific expression**	**1.0%**
16	Adenoma	No loss	(−)	Focal positive	(−)	Nonspecific expression	2.0%

Bold represents tumors classified as being cancer-type atypical parathyroid tumors based on the clustering analysis.

The median follow-up duration for patients was 32.0 (30.0–42.3) months and none of the 16 patients experienced biochemical recurrence, defined as re-elevation of calcium and/or PTH levels after initial normalization. However, patient 15, classified as having a cancer-type APT, experienced a persistent PTH elevation after a left superior parathyroidectomy. Diagnosed with PHPT at the age of 31, with a corrected calcium level of 11.1 mg/dL and PTH level of 827.0 pg/mL, she underwent parathyroidectomy after which her PTH level dropped to 19.8 pg/mL immediately post-surgery. To prevent hungry bone syndrome, postoperative supplementation with cholecalciferol (2,000 IU/day), calcium carbonate (2,500 mg/day) and calcitriol (0.5 μg/day) was administered. Nevertheless, her PTH level steadily increased, reaching 189.0 pg/mL at 2 months post-surgery. Her calcium levels remained stable around 9.0 mg/dL with a 25OHD of 14.2 ng/mL. Despite the correction for a vitamin D deficiency (corrected to 41.0 ng/mL), her PTH level was consistently around 90 pg/mL. Despite the increase in her PTH level, no definitive recurrent lesions have been identified on F-18 fluorocholine positron emission tomography-computed tomography and her calcium levels have remained stable. Therefore, she is being closely monitored with regular PTH and calcium assessments.

## Discussion

In this study, we demonstrated that our clustering model can effectively classify APTs based on their malignant potential, distinguishing them into either cancer-type or adenoma-type tumors. Furthermore, these classification results align well with the clinical manifestations.

The prognosis and clinical course of APT is still not fully understood. Several studies have suggested a benign course without recurrence, supporting the idea that long-term follow-up may not be necessary (McCoy *et al.* 2015, Galani *et al.* 2021). In two recent studies, the majority of patients did not experience recurrence during the follow-up period and were considered cured after surgery (Saponaro *et al.* 2021, Pardi *et al.* 2024). Similarly, in our cohort of 16 patients, no definite evidence of recurrence has been observed to date. However, a major limitation of the existing literature is the relatively short follow-up duration and small sample sizes. Considering that case reports have documented recurrences even at 10 years after surgery, as well as the potential contribution of small sample sizes to the scarcity of documented recurrences, we believe that reaching premature conclusions about APTs having a benign course should be avoided. Although complete resection of APTs may significantly reduce the risk of recurrence, these tumors are, by definition, characterized by worrisome histologic features that do not meet the full criteria for carcinoma but still raise concern for aggressive biological behavior (Erickson *et al.* 2022). Furthermore, emerging molecular studies suggest that some APTs share genomic features with parathyroid carcinomas, particularly in the presence of CDC73 alterations, supporting the idea that APTs may fall somewhere between benign and malignant parathyroid tumors rather than being a clearly separate category (Pardi *et al.* 2024). Therefore, we believe it is important to recognize the uncertain malignant potential of APTs when discussing prognosis and follow-up strategies. There is undoubtedly a need to develop an effective risk stratification model for APTs.

As our previous clustering model demonstrated the ability to effectively distinguish between parathyroid cancers and adenomas, we employed this model to classify APTs into cancer-type versus adenoma-type tumors in this study. Of the 16 tumor samples, four were classified as being cancer-type APTs, while 12 were classified as being adenoma-type APTs. The DEG expression patterns and gene ontology enrichment analyses revealed that each APT subtype exhibited strong transcriptomic similarity to its corresponding classical tumor type, further supporting the biological validity of this classification.

To further validate this classification, we analyzed the clinical manifestations of each group. Although clinical manifestations are not definitive criteria for discriminating parathyroid cancers from adenomas, severe hypercalcemia, markedly elevated PTH levels and young age can raise suspicion for parathyroid cancer (Betea *et al.* 2015). In line with this finding, we observed that patients with cancer-type APTs exhibited higher levels of calcium and PTH than those with adenoma-type APTs. The levels of calcium and PTH were highest in patients with cancer-type APTs, followed by those with adenoma-type APTs and then those with adenomas (reference group). Similarly, the age at diagnosis was the lowest for patients with cancer-type APTs, followed by those with adenoma-type APTs and then those with adenomas (reference group). This trend is consistent with previous observations that younger patients with PHPT tend to present with more pronounced biochemical abnormalities and more severe clinical features compared to older patients (Roizen & Levine 2012). Furthermore, patients with cancer-type APTs had lower BMDs and a larger size of tumors than the other groups. As the combination of a young age, elevated calcium and PTH levels, and a large tumor size are key factors for suspicion of parathyroid cancer (Betea *et al.* 2015), this finding demonstrates the potential effectiveness of our clustering model.

No recurrences were observed among our study participants. However, one patient with a cancer-type APT exhibited persistently high PTH levels after parathyroidectomy. Although her calcium levels remained stable and no definitive residual lesions were detected on imaging studies, the persistently elevated PTH levels and the cancer-type classification of her tumor certainly warrant long-term follow-up.

In earlier studies, IHC staining has been employed to distinguish parathyroid cancers from parathyroid adenomas (Juhlin *et al.* 2010, Kruijff *et al.* 2014). Parafibromin, the most well-known marker for parathyroid cancer (Cetani *et al.* 2013), is encoded by the *CDC73* gene and serves as a tumor suppressor protein. In patients with parathyroid cancer, the presence of a *CDC73* mutation and parafibromin loss has been associated with a low survival rate at 10-year follow-up (Cetani *et al.* 2013). The use of this marker in APT has also been investigated, revealing that a loss of parafibromin is associated with a higher recurrence rate in patients with APT (Kruijff *et al.* 2014). In accordance with these findings, the 2022 WHO guidelines suggest that parafibromin staining can be used for risk stratification of APTs, wherein a loss of parafibromin indicates a higher risk of recurrence (Erickson *et al.* 2022). In this study, a loss of parafibromin staining was observed in only one of the 16 patients. This patient (patient 9) had been diagnosed with PHPT at the age of 29 and underwent parathyroidectomy. Our clustering analysis classified his tumor tissue to be a cancer-type APT.

However, the clinical application of parafibromin immunostaining is challenging due to its variable sensitivity and interpretative complexity, limiting its utility for risk stratification in routine practice (Gill *et al.* 2019). Other markers, such as galectin-3, PGP9.5 and Ki67 have also been studied as potential markers of parathyroid malignancies. However, none of these markers have demonstrated satisfactory sensitivities or specificities. As no single marker has proven to be superior, a combination of these markers may be more effective (Truran *et al.* 2014). Similarly, the clinical utility of IHC markers for APT remains uncertain. In this study, we did not observe any valuable IHC marker findings, underscoring the need to identify novel markers.

In our previous study, we suggested that WT1 may be a potential marker for *CDC73*-mutant parathyroid cancer (Jo *et al.* 2023). WT1 inhibits *CDC73* while promoting the expression of MYC and BCL-2, facilitating cell proliferation and tumorigenesis (Rather *et al.* 2014). In addition, WT1 has been recognized as a potential biomarker for other cancers owing to its consistent upregulation in tumor tissues (Coosemans *et al.* 2007, Sera *et al.* 2008). While WT1 was originally characterized as a tumor suppressor gene, emerging evidence indicates that it may also act as an oncogene depending on the context, particularly in cases where gain-of-function mutations lead to aberrant activation of oncogenic pathways (Huff 2011). Conversely, loss-of-function mutations in WT1 have been reported in hematologic malignancies, suggesting a dual role in cancer pathogenesis. Notably, patient 9, whose tumor was a cancer-type APT with a double somatic *CDC73* mutation, exhibited positive WT1 staining as well as a loss of parafibromin. Although the pathological diagnosis indicated APT, the clustering analysis placed him within the cancer-type group. He presented with suspicious characteristics, including a young age at diagnosis, male gender and elevated levels of calcium and PTH. These observations support the clinical relevance of both our clustering model and WT1 staining, highlighting the need for closer follow-up for potential recurrences in these patients than others with APT.

This study has several strengths. While previous studies have primarily provided observational follow-up data on APTs, our study applied a molecular subtyping model to better assess their malignant potential. Given the current lack of clear guidelines for the prognosis and monitoring of APTs, this subtyping approach may serve as a useful tool to support clinical decision-making regarding long-term follow-up. In addition, we strengthened the clinical relevance of our findings by integrating transcriptomic data with detailed clinical characteristics and comparing these with a large, well-characterized reference cohort of patients with parathyroid adenomas.

This study had several limitations. To evaluate the validity of this clustering system, it would be ideal to compare the clinical course of patients using the long-term follow-up data. However, the rarity of APTs results in a limited sample size and the relatively short follow-up period further complicates drawing definitive conclusions about patient outcomes. Collecting such rare and well-documented cases remains a significant challenge. Moreover, WES was not performed on tumor or germline DNA, which may have limited the scope of genomic insights gained from this study. Future studies incorporating WES on both tumor and matched germline DNA would be valuable to provide a more comprehensive understanding of the genomic landscape of APTs. In addition, the clustering model used in our previous study was based on bulk RNA sequencing, which may not fully capture the gene expression profiles of parathyroid cancer. The current study also relied on bulk RNA sequencing, potentially limiting our understanding of gene expression in both parathyroid cancers and APTs. To address these limitations, future investigations should involve larger patient cohorts, longer follow-up periods and more advanced sequencing techniques to provide a more comprehensive assessment of gene expression in these conditions.

The genetic insights into APT have remained largely unclear. The clinical challenge lies in predicting the malignant potential of APTs and providing a proper management plan. In this study, we utilized transcriptome analysis to demonstrate an effective evaluation method for identifying the malignant potential of APTs. We believe our findings have the potential to support guidelines for the management and monitoring of APTs, which have been debatable until now. Further research involving larger cohorts and longer follow-up periods is warranted to enhance our understanding of the prognosis and optimal management strategy of patients with APT.

## Supplementary materials



## Declaration of interest

The authors declare that there is no conflict of interest that could be perceived as prejudicing the impartiality of the work reported.

## Funding

This study was supported by a Hur Won & Lee Eun Jig Research Grant of Yonsei University College of Medicine10.13039/501100008005 (6-2024-0043). It was also supported by the Se-jong Science Fellowship, National Research Foundation of Korea (NRF)10.13039/501100003725 grant funded by the Korea government (MIST) (RS-2024-00357369).

## Author contribution statement

H-S P, J J J, N H and Y R contributed to the research idea and study design. H-S P, M K, S-Y J and Y R contributed to data analysis/interpretation. G J K, S K and Y R were responsible for supervision and mentorship.

## Data availability

The data generated in this study are publicly available in NCBI SRA at PRJNA1184316.
