# Supplementary material for: Transcriptome sequencing reveals distinct atypical parathyroid tumor subtypes
Source: Endocr Relat Cancer. 2025 Jun 14;32(6):e250057. doi: 10.1530/ERC-25-0057 (PMC12170948; doi:10.1530/ERC-25-0057)

**Supplementary Table 1. Diagnostic criteria of atypical parathyroid tumors identified among study participants**

| Patient No. | Tumor cells in capsule | Trabe-cular/ solid growth | Cellular nests in a thickened connective tissue | Band like fibrosis | Diffuse sheet-like monotonous small cells with high N/C ratio | Diffuse cellular atypia | Macro-nucleoli present in many tumor cells | Adherence to adjacent structures without frank invasion | Atypical mitotic figures | Coagulative necrosis | Increased mitotic activity (>5/10mm$^2$) | No. of criteria met |
|---|---|---|---|---|---|---|---|---|---|---|---|---|
| **1** | (+) | (+) | (+) | (+) | (-) | (-) | (+) | (-) | (-) | (-) | (-) | 5/11 |
| **2** | (+) | (+) | (+) | (+) | (-) | (-) | (-) | (+) | (-) | (-) | (-) | 5/11 |
| **3** | (+) | (+) | (+) | (+) | (-) | (-) | (-) | (-) | (-) | (-) | (-) | 4/11 |
| **4** | (+) | (+) | (-) | (-) | (+) | (-) | (+) | (-) | (+) | (-) | (-) | 5/11 |
| **5** | (+) | (+) | (+) | (+) | (+) | (-) | (-) | (-) | (-) | (-) | (-) | 5/11 |
| **6** | (+) | (+) | (+) | (+) | (+) | (-) | (-) | (+) | (-) | (-) | (-) | 6/11 |
| **7** | (+) | (+) | (+) | (+) | (+) | (+) | (+) | (-) | (-) | (-) | (-) | 7/11 |
| **8** | (+) | (+) | (+) | (-) | (-) | (+) | (-) | (+) | (-) | (-) | (-) | 5/11 |
| **9** | (+) | (+) | (+) | (-) | (-) | (+) | (+) | (-) | (-) | (-) | (-) | 5/11 |
| **10** | (-) | (+) | (-) | (-) | (+) | (+) | (-) | (-) | (+) | (-) | (-) | 4/11 |
| **11** | (+) | (+) | (-) | (+) | (+) | (+) | (-) | (-) | (-) | (+) | (-) | 6/11 |
| **12** | (+) | (+) | (+) | (+) | (-) | (-) | (-) | (-) | (-) | (-) | (-) | 4/11 |
| **13** | (+) | (-) | (+) | (+) | (-) | (-) | (-) | (-) | (-) | (-) | (-) | 3/11 |
| **14** | (+) | (+) | (+) | (+) | (+) | (+) | (-) | (-) | (-) | (-) | (-) | 6/11 |
| **15** | (+) | (+) | (-) | (+) | (-) | (+) | (+) | (-) | (+) | (-) | (-) | 6/11 |
| **16** | (+) | (-) | (+) | (+) | (-) | (-) | (-) | (-) | (-) | (-) | (-) | 3/11 |

*Note:* Gray represents tumors classified as being cancer-type atypical parathyroid tumors based on the clustering analysis. (+) indicates positive and (-) indicates negative.

N/C ratio, nuclear-cytoplasmic ratio

**Supplementary Table 2. Correlation and regression analysis of serum PTH and BMD by site**

| | Pearson correlation | | Linear regression | |
|---|---|---|---|---|
| | **R** | **P-value** | **β** | **P-value** |
| **Lumbar spine** | -0.076 | 0.223 | -0.00042 | 0.223 |
| **Femoral neck** | -0.159 | 0.01 | -0.00063 | 0.01 |
| **Total hip** | -0.189 | 0.002 | -0.00074 | 0.002 |
| **One-third radius** | -0.4 | <0.001 | -0.0029 | <0.001 |

PTH, parathyroid hormone; BMD, bone mineral density.

**Supplementary Figure 1. Representative histologic images of atypical parathyroid tumors**

A. Atypical parathyroid tumors exhibit atypical histologic features including enlarged hyperchromatic pleomorphic cells (Patient 7) (H&E, 100×, original magnification). B. Presence of band-like fibrosis (Patient 5) (H&E, 40×, original magnification). C. Tumor revealing areas of monotonous cells with a solid growth pattern (Patient 10) (H&E, 100×). D. Tumor cell nests in thick fibrous capsule (Patient 9) (arrows, H&E, 40×, original magnification).

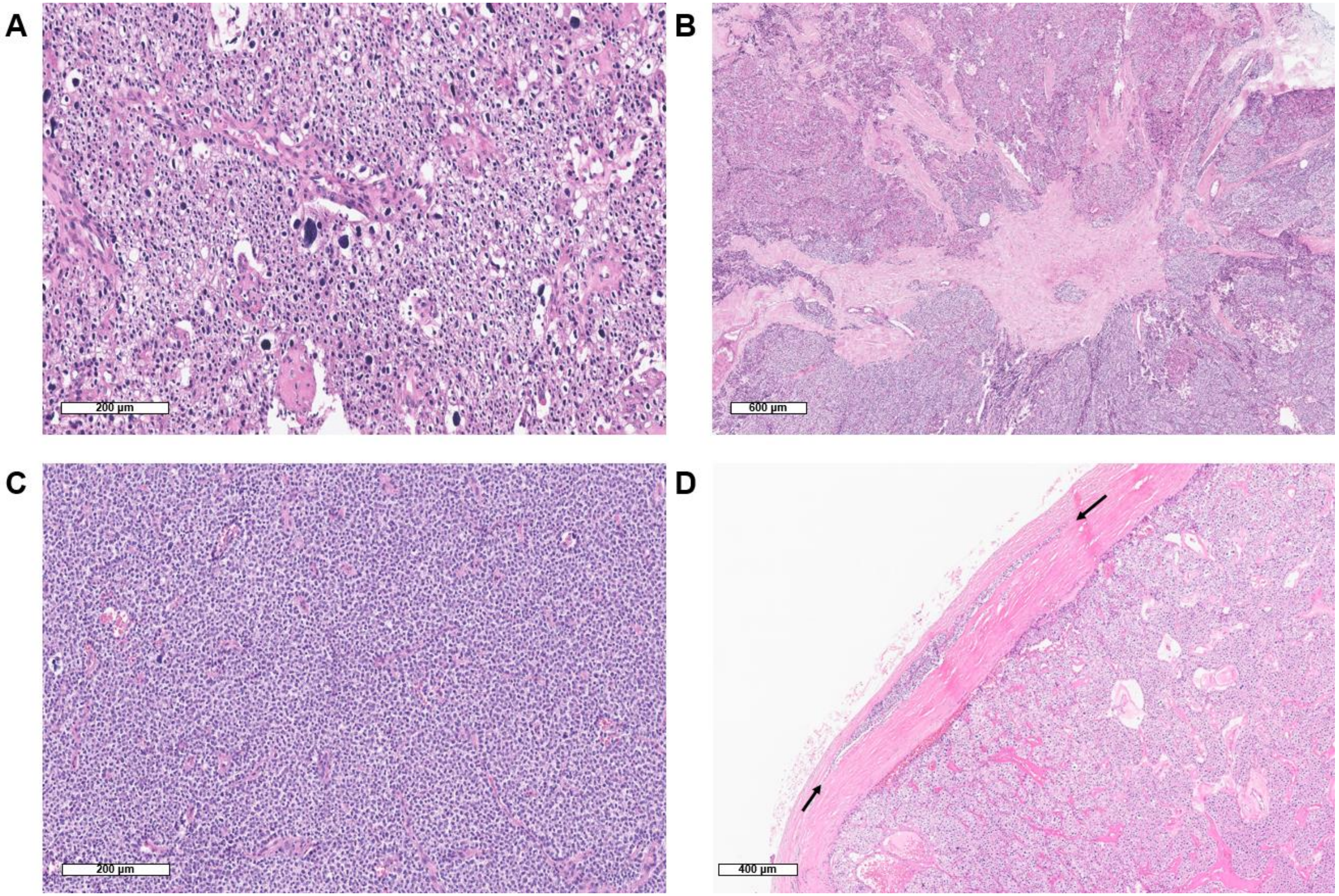

**Supplementary Figure 2. Comparison of gene expression between APT subtypes and classical parathyroid tumors**. Scatter plots showing the expression patterns of DEGs in APT subtypes and their corresponding classical tumor types. (A) Expression levels of DEGs in adenoma-type APTs versus classical adenomas. (B) Expression levels of DEGs in carcinoma-type APTs versus classical carcinomas. High concordance was observed (R = 0.87 and 0.84, respectively), supporting the transcriptional similarity between APT subtypes and the classical parathyroid tumors.

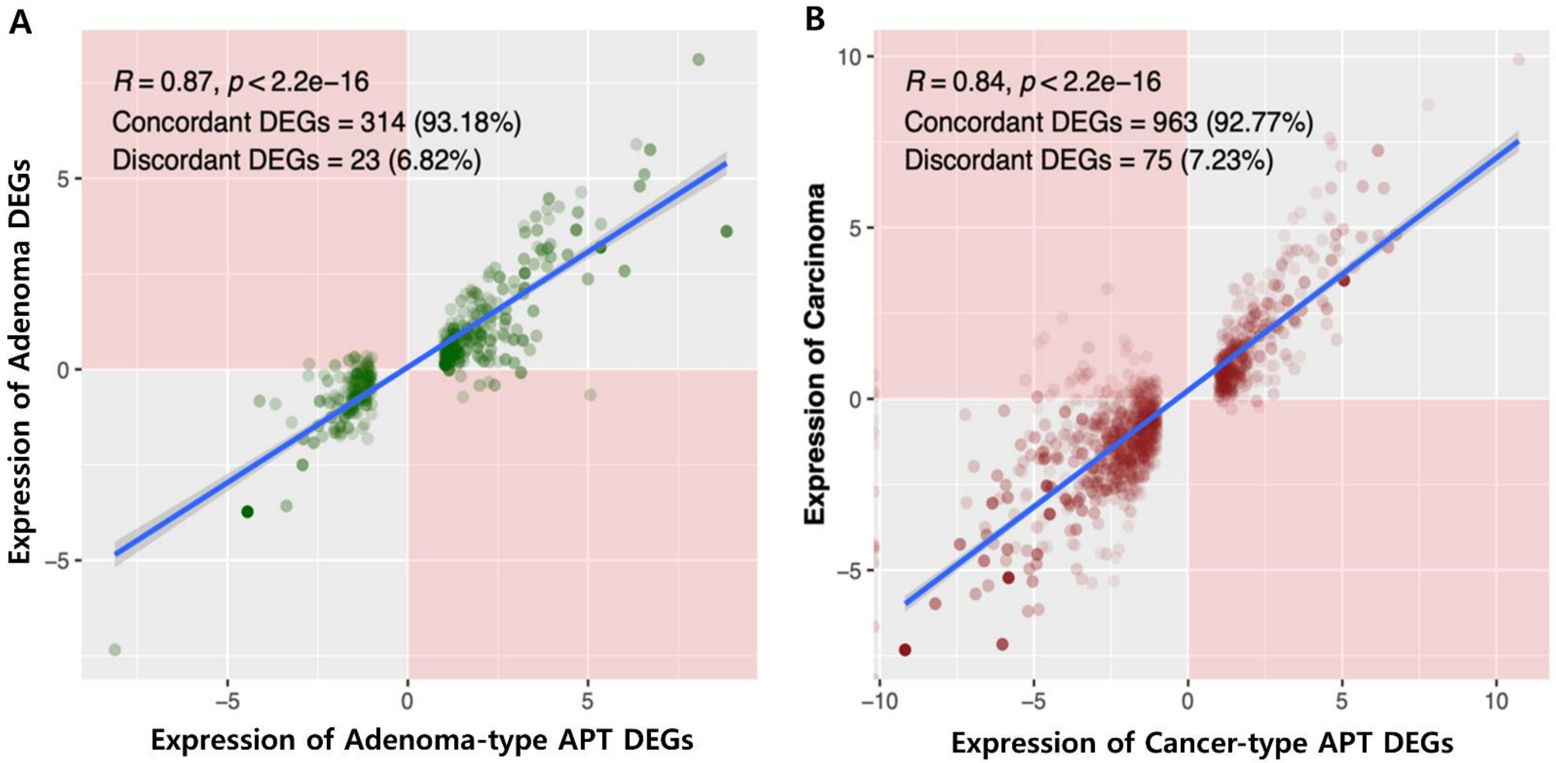


APT, atypical parathyroid tumor; DEG, differentially expressed gene.

**Supplementary Figure 3. GO enrichment analysis of DEGs in APTs and classical parathyroid tumors.** (A) Significantly enriched GO terms identified from DEGs in adenoma and adenoma-type APTs. (B) Significantly enriched GO terms identified from DEGs from carcinoma and carcinoma-type APTs. Dot size represents the proportion of genes involved in each term (GeneRatio), and color indicates the adjusted p-value.

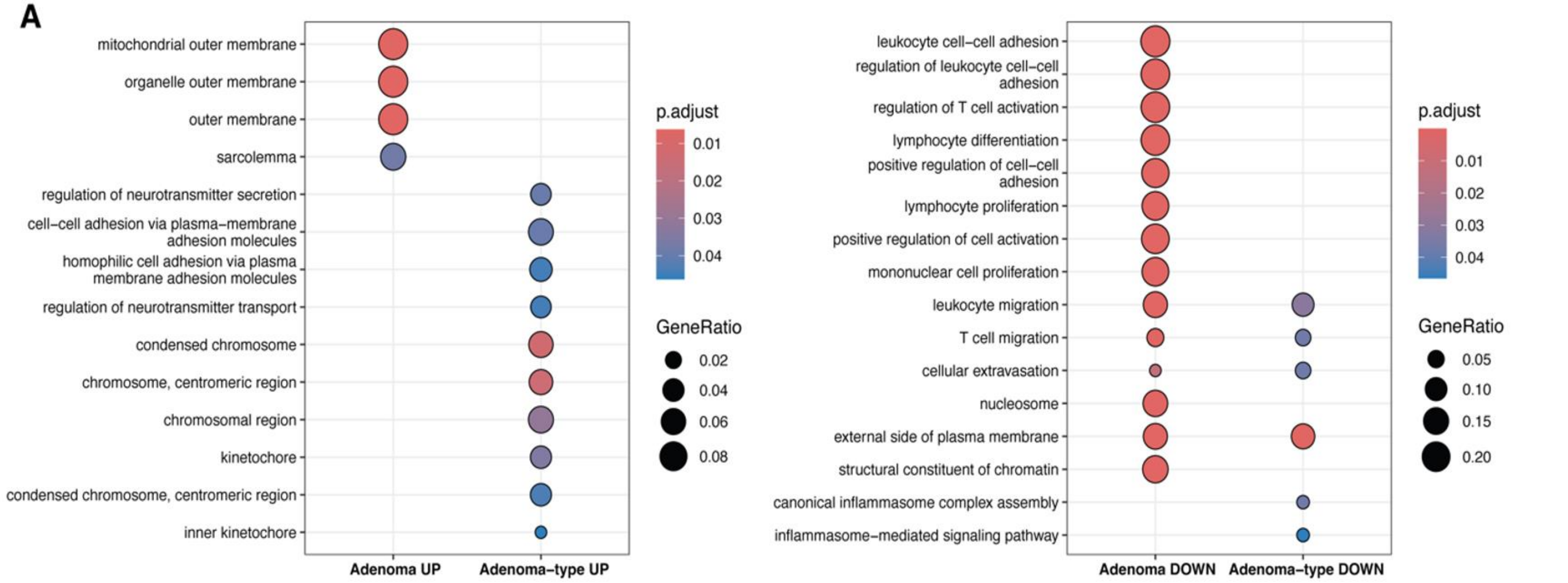

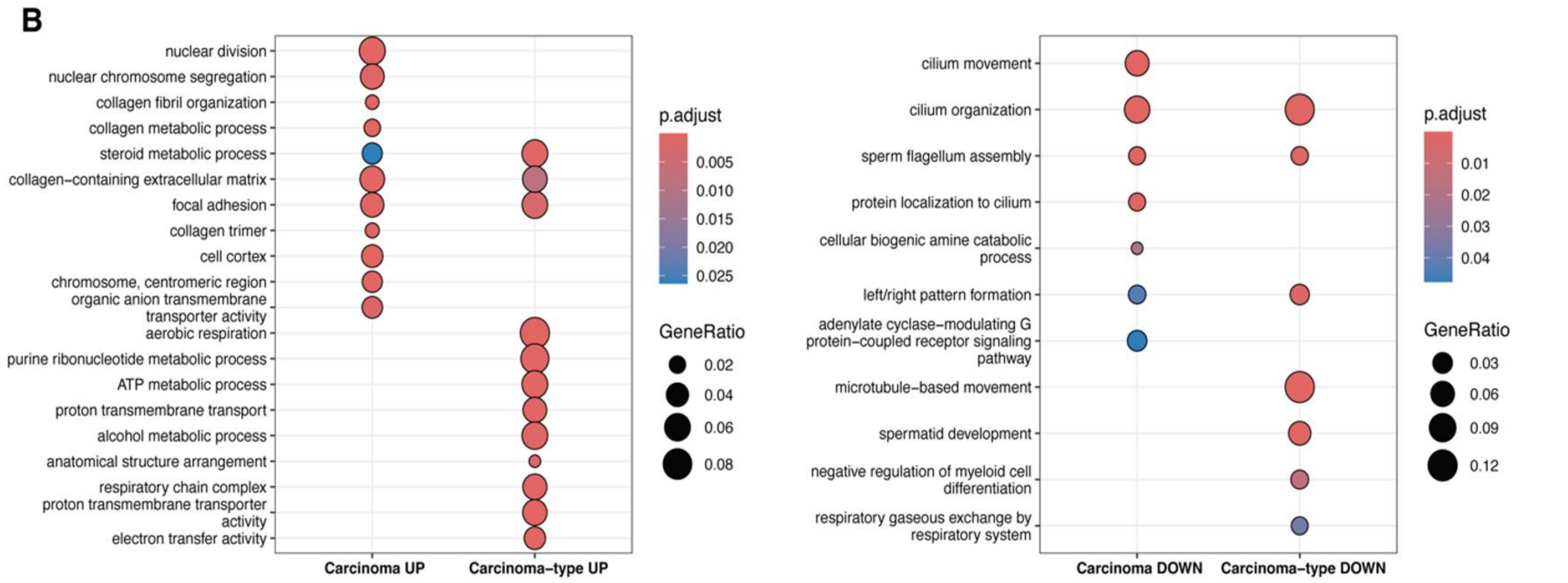


GO, gene ontology; DEG, differentially expressed gene; APT, atypical parathyroid tumor.

**Supplementary Figure 4. Correlation between tumor size and PTH levels across pathology groups.** Scatter plot illustrating the relationship between the longest diameter of parathyroid tumors and serum PTH levels. Red, Cancer-type APTs; Green, adenoma-type APTs; Blue, reference group with parathyroid adenomas.

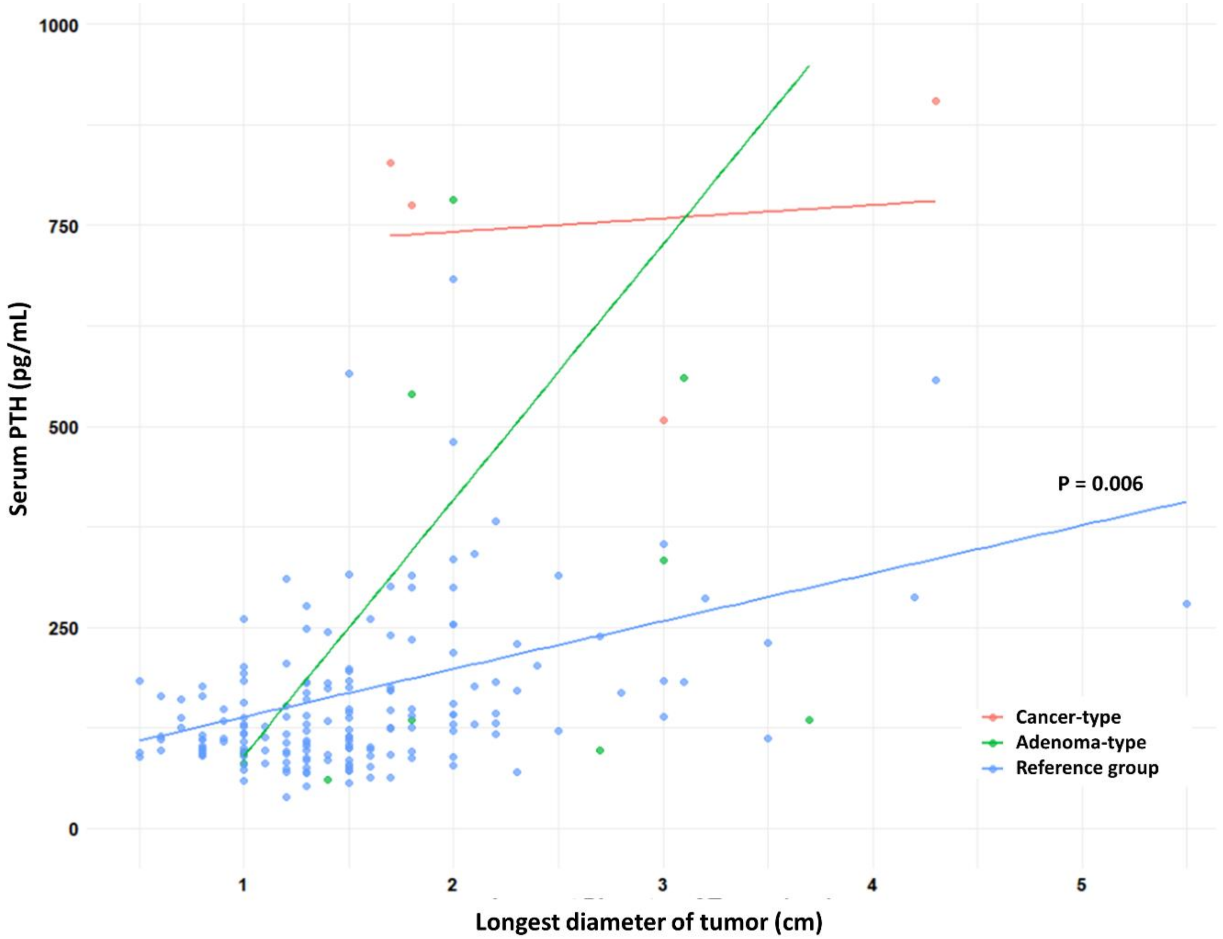


PTH, parathyroid hormone; APT, atypical parathyroid tumor.

**Supplementary Figure 5. Representative images of immunohistochemical expression of WT1 and parafibromin in atypical parathyroid tumors**

A. Moderate-to-strong nuclear expression in ≥5% of tumor cells were considered positive (200×, original magnification, Patient 9). B. Staining in <5% cells with weak nuclear expression was regarded as negative (200×, original magnification). C. Complete loss of parafibromin expression was observed in one case (200×, original magnification, Patient 9). D. The remaining cases exhibited no loss of parafibromin expression (200×, original magnification).

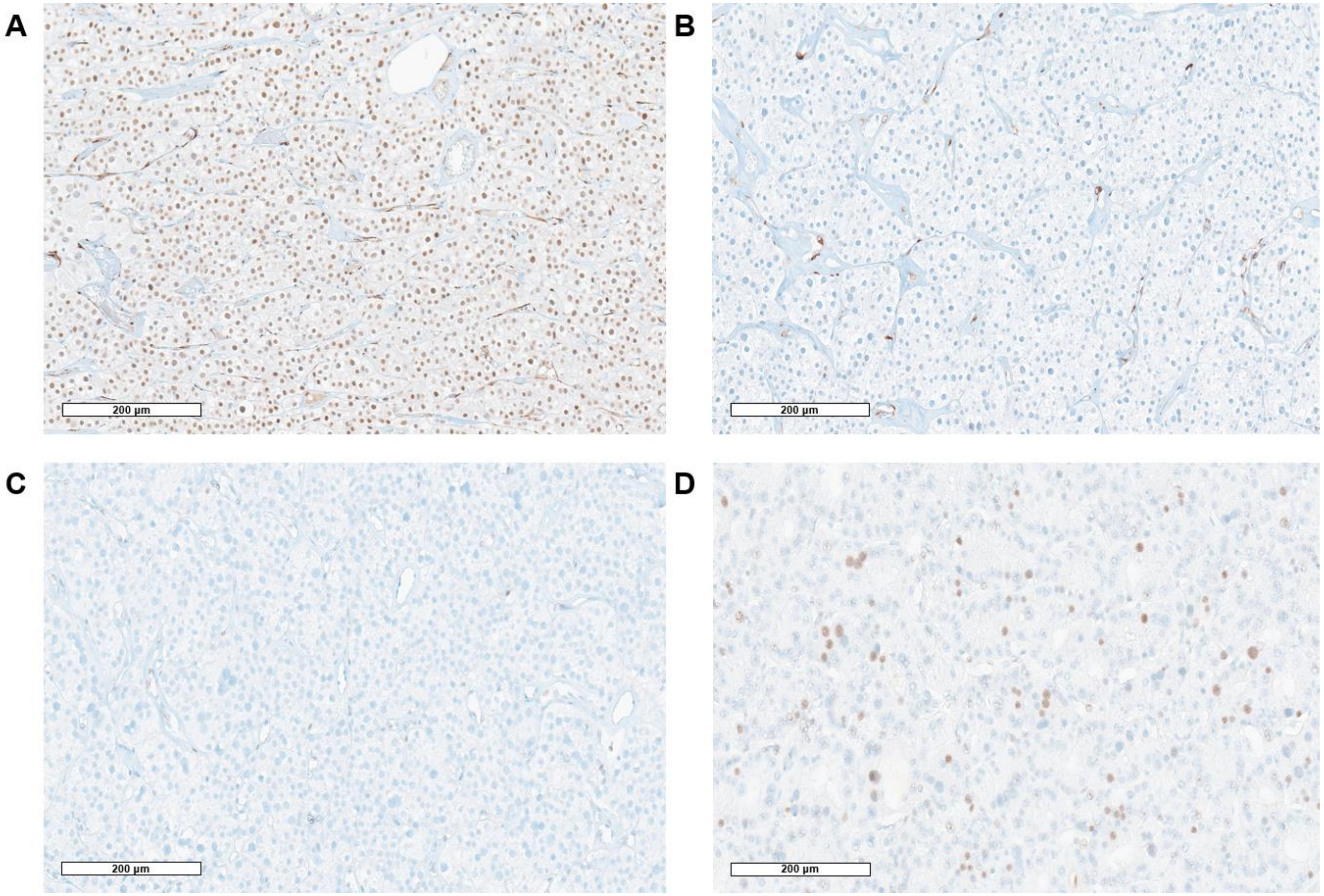

Supplement: Supplementary file 1 [file supplementary_materials.pdf]
